# Evaluation of the efficacy of Conbercept in the treatment of diabetic macular edema based on OCTA

**DOI:** 10.1097/MD.0000000000021992

**Published:** 2020-08-28

**Authors:** Teer Ba, Lili Zhou, Han Zhang, Xiaoguang Zhang, Shixuan Guo, Huixia Li, Haiyan Tian, Qiqige Caihan, Gang Bai, Jing Zhou, Lao Qi, Xueyan Zhang, Guisen Zhang

**Affiliations:** a School of Public Health, Inner Mongolia Medical University, Hohhot, China; b Inner Mongolia Chaoju Eye Hospital, Hohhot, China.

**Keywords:** clinical trial, conbercept, diabetic macular edema

## Abstract

**Background:**

Diabetic macular edema (DME) can cause severe vision impairments for patients with diabetes. Recently, Conbercept has shown efficacy on DME with 3-monthly loading dose injection and pro re nata (PRN, 3+PRN) thereafter in retrospectivetrials. Furthermore, there are some other approaches have been recommended such as 2mg bimonthly (2q8) after 5 initial doses, or Conbercept 0.5mg treat-and-extend, however, some patients still have recurrence of the disease after treatment. Therefore, in order to identify more efficacy and safety approach on Conbercept inpatients with DME, a randomized controlled trial will be performed with 6-monthly loading dose injection and PRN (6+PRN) compared with 3+PRN treatments.

**Methods:**

This study is a multicenter, randomized control trial of Conbecept treating DME in China. Patients with type 2 diabetes suffered from DEM who already planned to receive Conbercept treatment will be recruited. All subjects will be randomized divided into either a study agent treatment group (6+PRN) or a control group (3+PRN), and observes the subjects for 48 weeks after initiation of treatment.

**Results:**

This study will provide a new powerful evidence of the efficacy and safety of Conbecept treating DME.

**Discussion:**

This RTC study will determine whether multiple treatments of Conbercept provide better effectiveness in patients with DME.

**Trial registration number:**

ChiCTR2000032728

## 1 Introduction

Diabetic retinopathy (DR) was the most common cause of bilateral blindness in working-age population in the world.^[1–3]^ Diabetic macular edema (DME) is one of the important causes of visual impairments caused by DR. A recent systematic review and meta-analysis showed that DME were prevalent in 3.7% (95% CI 2.2–6.2%). The pooled mean annual incidence of DME in persons with type 2 diabetes was 0.4% (95% CI 0.5–1.4%). It is estimated that persons with diabetes affected by DR and DME in Europe will be up to 8.6 million in 2050, of whom 30% require treatment.^[4]^ Vascular endothelial growth factor (VEGF) is an important factor in the destruction of the blood-retinal barrier, which can lead to blood vessel leakage and macular edema.^[5]^ The level of VEGF in the eyes of DME patients increases, so it is proposed that the use of anti-VEGF (anti-VEGF) drugs may control the process of vision loss or even loss in DME patients.

Conbercept is a new fusion protein, which contains Fc segments of human VEGF receptor 1, VEGF receptor 2, and human IgG-1. It can be combined with VEGF-A, VEGF-B, VEGF-C, and placental growth factor (PlGF).^[6–8]^ In May 2019, Conbercept had been approved to use for the treatment of the DME by China Food and Drug Administration.

Previously, a retrospective study evaluated the therapeutic efficacy of conbercept for treatment of DME patients received one initial intravitreal injection of Conbercept (IVC) followed by retreatment based on best-corrected visual acuity (BCVA) loss or central subfield thickness (CST) increase and found that the mean improvement in BCVA was significantly greater in the Conbercept-treated groups than that of the corresponding untreated controls at 12 months. In addition, the mean CST was significantly reduced in the Conbercept treatment groups as compared to that of the corresponding untreated controls. Furthermore, another retrospective clinical study showed that DME patients initially treated with 1 to 3 consecutive monthly IVC injections could be effective for visual and anatomic improvements.^[9,10]^ However, there is no report from randomized controlled trial based evidence on IVC for DME. In addition, there are some regimens of intravitreal anti-vascular endothelial growth factor (VEGF) treatment on DME such as Treat-and-extend or pro re nata (PRN; “as needed”) regimens have been found to reduce the injection burden on patients.^[11]^ In clinic, patients with DME often require multiple treatments, and some patients have poor or even no response to the drugs, thus, there is a need to conduct a randomized controlled trial study to identify a more effectiveness approach on IVC for DME.

This aim of this study was to assess the safety and efficacy of 6 + PRN therapy with anti-VEGF agent, Conbercept in patients with T2DM and DME, and to compare the traditional 3+ PRN regimen.

## 2 Subjects and methods

Ethical approval for this study was granted by the Office for Research Ethics Committees of Hohhot Chao Ju Eye Hospital ([2020] KY0501). This study protocol was registered at the Chinese clinical trial registry (Chictr.org.), ChiCTR200032728.

### 2.1 Study design and setting

This is a multicentre, randomised, equivalence, single-masked clinical trial set within specialized Eye Hospitals involving Hohhot Chao Ju Eye Hospital, Chifeng Chao Ju Eye Hospital, Baotou Chao Ju Eye Hospital, Datong Chao Ju Eye Hospital, Jining Chao Ju Eye Hospital and Bayannur Disabled Persons’ Federation Eye Hospital in China. It aims to evaluate the clinical effectiveness and safety of 6 + PRN IVC, when compared with 3 + PRN IVC, for the treatment of patients with DME.

### 2.2 Participants

Potential study participants will be identified through comprehensive ophthalmic examination. Informed consent will be given to the eligible participants and they will be given time to think about their participation and ask questions about the study; if approved, the participant will be recruited into this study.

### 2.3 Inclusion criteria

Eligible participants will have Type 2 diabetes and DME, as determined by SD-OCT, in 1 or both eyes with either:

CST of > 300 microns as determined by SD-OCT due to DME or

CST of < 300 microns provided that intraretinal and/or subretinal fluid is present in the central subfield (central 1 mm) related to DME

Have visual acuity of > 24 early treatment diabetic retinopathy study (ETDRS) letters (Snellen equivalent > 20/320)

Be amenable to laser treatment, as judged by the treating ophthalmologist

Be over 18 years of age

### 2.4 Exclusion criteria

Ineligible for IVC treatment, as judged by the treating ophthalmologist

Has severe PDR requiring surgery

Has received intravitreal anti-VEGF therapy or steroids within the previous 3 months

Has received retinal laser treatment within the previous 3 months

Has had cataract surgery within the previous 6 weeks

Has glaucoma, retinal vein occlusion, uveitis, optic nerve disease, or other fundus diseases except DME

Has severe cardiovascular, cerebrovascular, liver, and hematopoietic system diseases, and diabetic nephropathy with renal failure

Has serious life-threatening primary disease or mental illnesse

Are pregnant or breastfeeding

Are participating in clinical trials of other drugs

### 2.5 Outcome measures

#### 2.5.1 Primary outcome

Mean change in BCVA on the study eye at both 24 and 48 weeks following treatment

#### 2.5.2 Secondary outcomes

Mean change in CST in the study eye from baseline to both 24 and 48 weeks

Mean change in retinal microvascular metrics by optical coherence tomography angiography (OCTA)

Occurrence in the percentage (%) of side effects from baseline to 48 weeks

Number of injections performed

Use of additional treatments

### 2.6 Randomization

All participants will be randomised 1:1 using computer-generated randomization, with sequentially numbered, to receive either 3 + PRN or 6 + PRN treatment. All participants are single-masked of the allocation and regimen after randomization. Randomization is advised to be conducted on the day of IVC treatment.

### 2.7 Interventions

All patients in 3 + PRN group will receive a minimum of 3 initial monthly IVC (0.5 mg) injection, PRN dosing thereafter BCVA and CST stabilization criteria-driven PRN treatment while 6 + PRN group will receive a minimum of 6 initial monthly IVC (0.5 mg) injection, PRN dosing thereafter BCVA and CST stabilization criteria-driven PRN treatment.

### 2.8 Study procedures

#### 2.8.1 Patient evaluation

All participants will be assessed from baseline to 48 weeks according to the schedule of assessments (Table 1). Primary outcome, BCVA will be measured ETDRS visual acuity charts at 4 m at baseline and at months 1, 3, 6, and 12. Secondary outcomes, CST will be measured ETDRS visual acuity charts at 4 m at baseline and at months 1, 3, 6, and 12, while OCTA will be checked at 6 and 12 months.

**Table 1:**
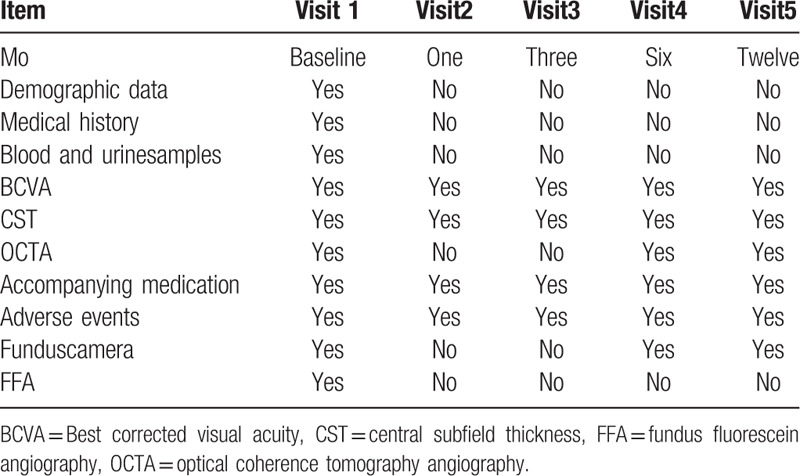
Assessment protocol for the current study.

#### 2.8.2 Data collection

Case report form (CRF) will be used to collect data for this study.

### 2.9 Quality control

(1) Observation ophthalmologists will receive pre-clinical training, have a full understanding of the specific content of the clinical trial program, and check and record according to the time and method specified in the program.
(2) Special personnel regularly check the progress of clinical trials, and convene clinical interim meetings to ensure that trials are conducted in accordance with the protocol, drug clinical trial management regulations, and related regulations.
(3) The inspection center and other relevant departments establish standard operating procedures and quality control procedures.
(4) Establish a test coordination committee. The coordination committee is responsible for the implementation of the entire test and studies and solves the problems related to the test.

### 2.10 Sample size

This study is powered to demonstrate non-inferiority of 6 + PRN with respect to the effective rate (at 48 weeks). The trial will have sufficient statistical power to determine superiority of 6 + PRN treatment over the 3 + PRN. According to our clinical application, we initially expect the effective rate of IVC for DME to be around 75%. After calculation, 50 cases are required for each group. Considering the shedding rate of 20%, 60 cases are required for each group, and a total of 120 cases for the 2 groups.

### 2.11 Statistical analysis

SPSS statistical software (IBM Statistics, v. 22.0; IBM Corp, Armonk, NY) will be used to conduct analysis. Quantitative data, BCVA, is represented by median (quartile), and other data is represented by mean and SD. The comparison of clinical features between groups was performed using the *t* test. Changes in BCVA before and after treatment were tested using Wilcoxon–Matt–Whitney test. The change of CST and indicators of retinal blood flow from baseline were analyzed by paired *t* test. The comparison of different follow-up time indicators was performed by repeated measurement data analysis of variance. Statistical significance will be based on 2-sided tests, with *P* value less than .05 taken as the criterion for statisticalsignificance.

## 3 Discussion

Currently, this will be the first study to evaluate the effectiveness and safety of 6 + PRN regimen IVC compared with 3 + PRN regimen. Progress made so far (till July 6, 2020): recruitment has started at all participating sites (n=7). To date, a total of 28 eyes have been recruited into the trial.

## Author contributions

**Funding acquisition:** Lao Qi.

**Investigation:** Huixia Li.

**Project administration:** Teer Ba, Xiaoguang Zhang.

**Resources:** Han Zhang, Qiqige Caihan.

**Software:** Shixuan Guo.

**Supervision:** Jing Zhou.

**Validation:** Haiyan Tian.

**Writing – original draft:** Lili Zhou, Xueyan Zhang.

**Writing – review & editing:** Guisen Zhang.
